# Karyotype and chromosome banding of endangered crucian carp, *Carassius carassius* (Linnaeus, 1758) (Teleostei, Cyprinidae)

**DOI:** 10.3897/CompCytogen.v7i3.5411

**Published:** 2013-08-23

**Authors:** Martin Knytl, Lukáš Kalous, Petr Ráb

**Affiliations:** 1 Department of Zoology and Fisheries, Faculty of Agrobiology, Food and Natural Resources, Czech University of Life Sciences Prague, 16521 Praha 6 - Suchdol, Czech Republic; 2 Laboratory of Fish Genetics, Institute of Animal Physiology and Genetics, AS CR v.v.i., 277 21 Liběchov, Czech Republic

**Keywords:** Fish cytogenetics, paleotetraploid, heterochromatin, metaphase chromosomes

## Abstract

The karyotype and other chromosomal characteristics the crucian carp (*Carassius carassius* (Linnaeus, 1758)) were revealed by means of conventional banding protocols (C, CMA_3_, AgNOR). The diploid chromosome number (2n) in this species was 100. Its karyotype was composed of 10 pairs of metacentric, 18 pairs of submetacentric and 22 pairs of subtelo- to acrocentric chromosomes without any microchromosomes. C-banding identified blocks of telomeric heterochromatin on seven chromosome pairs. The NORs were situated on the p arms of the 14^th^ pair of submetacentric chromosomes and on the p arms of the 32^nd^ pair of subtelo-acrocentric chromosomes; AgNOR-positive signals corresponded to the CMA_3_-positive signals. These chromosome characteristics may suggest a paleo-allotetraploid origin of *Carassius carassius* genome.

## Introduction

The crucian carp, *Carassius carassius* (Linnaeus, 1758), is a cyprinid fish that inhabits densely vegetated backwaters and oxbows of lowland rivers, shallow lakes and ponds. It is a native species to Europe with a distribution extending eastwards from the River Rhine to the River Kolyma in Siberia (Szczerbowski 2002, Kottelat and Freyhof 2007). Despite its ability of “tissue breathing” (Blažka 1958) which helps it to survive in unfavourable conditions, the crucian carp has undergone a substantial decline in many localities during the last decades (Navodaru et al. 2002, Kottelat and Freyhof 2007, Sayer et al. 2011). Indisputable disappearance from nature resulted in the inclusion of the crucian carp in the list of endangered species by authorities of several EU countries (Economidis 1995, Schiemer and Spindler 2006, Copp et al. 2008, Sayer et al. 2011).

There is a number of factors that may have contributed to the disappearance of *Carassius carassius*, including habitat loss and degradation (Copp 1991, Holopainen and Ikari 1992, Wheeler 2000), displacement via competition with introduced species such as the polyploid biotype of the Prussian carp *Carassius gibelio* (Bloch, 1782), the Amur sleeper *Perccottus glenii* (Dybowski, 1877), feral goldfish *Carassius auratus* (Linnaeus, 1758) and the common carp *Cyprinus carpio* (Linnaeus, 1758) (Tarkan et al. 2012, Litvinov and O’Gorman 1996, Copp et al. 2005, Lusk et al. 2010). Moreover, all species of *Carassius* Nilsson, 1832 present in Europe (Rylková et al. 2013), including the crucian carp (*Carassius carassius*), Prussian carp (*Carassius gibelio*), ginbuna (*Carassius langsdorfii* Temminck & Schlegel, 1846) and goldfish (*Carassius auratus*)are often confused due to their morphological similarity (Hensel 1971, Kalous et al. 2007). Such confusion may lead to inappropriate stocking of wrong species instead of intended support of a local endangered population of crucian carp with negative consequences (Sayer et al. 2011).

Genetic contamination seems to be a very important but hidden threat to *Carassius carassius* that has been recently discovered. Hybridization occurs between *Carassius carassius* and *Carassius gibelio* (Prokeš and Baruš 1996). This type of hybridization was later confirmed using molecular (Papoušek et al. 2008, Wouters et al. 2012) and cytogenetic techniques (Knytl et al. 2013) in Sweden and the Czech Republic. Hybrids between *Carassius carassius* and *Carassius auratus* (Hänfling et al. 2005, Smartt 2007) and intergeneric hybrids between *Carassius carassius* and *Cyprinus carpio* (Hänfling et al. 2005) were discovered in England also by using microsatellite analysis. We believe that these processes also take place in other localities where *Carassius carassius*, *Carassius auratus* and/or *Carassius gibelio* co-occur. Moreover, molecular data suggest that these hybrids are able to reproduce and form filial generations by backcrossing (Hänfling et al. 2005, Wouters et al. 2012).

The cytogenetics of *Carassius carassius* is still poorly understood, since only a few studies of this speciesbased onGiemsa-stained chromosomes are known (Table 1). Interestingly, two different diploid chromosome numbers 2n = 50 and 2n = 100 were reported.

**Table 1.** Chromosome numbers and karyotypes of *Carassius carassius* reported from Europe; NA = not available.

2n	Diploid karyotype	Locality	Source
104	20m+72sm+12a	NA	Chiarelli et al. 1969
100	20m+44sm+36a	France	Hafez et al. 1978
100	52m-sm+48 st-a	Drina R., Ukrinski Lug (Bosnia)	Sofradžija et al. 1978
100	20m+40sm+40a	the Netherlands	Kobayasi et al. 1970
50	20m+12sm+18s-ta	lower Danube R. (Romania)	Raicu et al. 1981
100	48m-sm+52st-a	Russia	Vasilev and Vasileva 1985
100	NA	Elbe R. System (Czech Republic)	Mayr et al. 1986
100	NA	Vistula R. System (Poland)	Boroń et al. 2010
100	20m+36sm+44st-a	Elbe R. System (Czech Republic)	This study

Such an unclear situation encourages us to present cytogenetic analyses of *Carassius carassius* with respect to ongoing hybridization processes and threats in European waters. The present study deals with chromosomal characteristics of crucian carp (*Carassius carassius*) from the locality Byšičky in vicinity of the Elbe River (Czech Republic). Prussian carp (*Carassius gibelio*) and crucian carp co-occur in this place and the a hybrid allopolyploid female with 206 chromosomes was recently discovered there (Knytl et al. 2013). In this paper, we have used Giemsa staining as well as banding techniques like C, CMA_3_, AgNOR and DAPI (4’, 6-diamino-2-phenylindole) banding.

## Material and methods

### Fish sampling

Four females and one male werecollected during a field survey of ichthyofauna in alluvial ponds and old oxbows of the Elbe River close to the city of Lysá nad Labem (GPS: 50°10.75' N, 14°47.62' E). All five individuals were identified morphologically as common *Carassius carassius* (not the dwarf form)according to Szczerbowski (2002) and Kottelat and Freyhof (2007). This material is deposited as voucher specimens in the collection of the Department of Zoology and Fisheries, Czech University of Life Sciences Prague under number KZR141083Cc.

### Chromosome preparation and staining

All collected fish were subjected to a non-destructive procedure for chromosome preparation from fin clips developed by Völker and Kullmann (2006) and modified by Kalous et al. (2010); 50 metaphases from each individual were analyzed. Metaphase chromosomes stained in 4 % Giemsa-Romanowski solution in phosphate buffer (pH = 7) were counted with PC software QuickPhoto Micro. Karyotypes were arranged using PC software Ikaros (karyotyping system), version V 3.4.0 and Adobe Photoshop, version CS7. Chromosome morphology was determined according to Levan et al. (1964). Analyzed slides with recorded co-ordinates of selected metaphases were cleaned in xylene for 2 minutes, then in benzoin for 2 minutes and finally destained in fixative (methanol: acetic acid; 3:1, v/v) for 3 minutes. Chromosome slides were then stored at +4°C for 12 hours before banding experiments. Chromosome banding (CMA_3_, DAPI, C and AgNOR) was carried out according to Rábová et al. (2013). Different slides were used for each banding method (non-sequential chromosome banding), except for the sequential DAPI + CMA_3_. Valid Animal Use Protocols were in force at the Institute of Animal Physiology and Genetics and Czech University of Life Sciences Prague during this study.

### Microscopy and image processing

CMA_3_, DAPI, C-banding and AgNOR images were captured with a cooled CCD camera Olympus DP30BW (equipped with a black-and-white (B&W) CCD-Chip Sony ICX285-AL) coupled to an epifluorescence microscope Olympus AX70 equipped with a set of 3 narrowband fluorescent filters. Micrographs were captured with the Olympus Acquisition Software and B&W images were processed with the software Micro Image. Altogether 200 images (metaphases), i.e. 50 images for each banding type (CMA_3_, DAPI, C and AgNOR) were taken and analyzed.

## Results

### Karyotype

**Figure 1.**
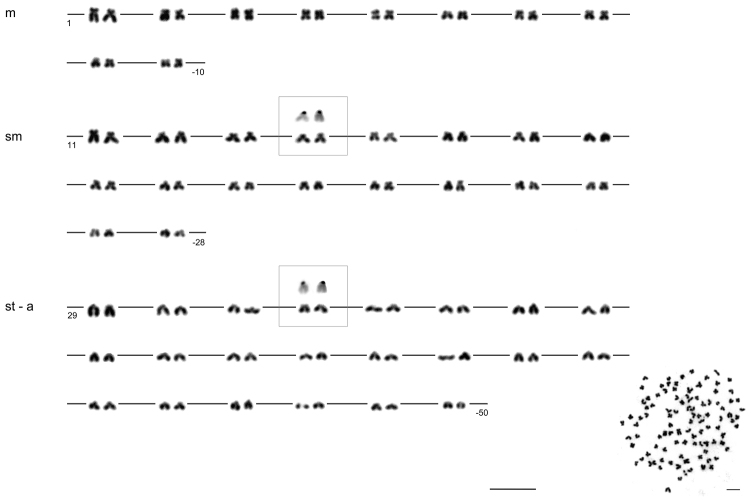
Karyotype of *Carassius carassius* female arranged from Giemsa-stained chromosomes (shown as inlay); m – metacentric, s – submetacentric, st – subtelocentric, a – acrocentric chromosomes. Four CMA_3_-positive (color-inverted) chromosomes (14^th^ pair of sm chromosomes and 32^nd^ pair of st-a chromosomes) are additionally shown in the frames. Bar = 10 μm.

The diploid chromosome number of the examined individuals was invariably 2n = 100 (75 % investigated metaphases). The karyotype consisted of 10 pairs of metacentric (m), 18 pairs of submetacentric (sm) and 22 pairs of subtelo- (st) to acrocentric (a) chromosomes without any microchromosomes (Fig. 1).

### Chromosome banding and AgNOR staining

**Figure 2.**
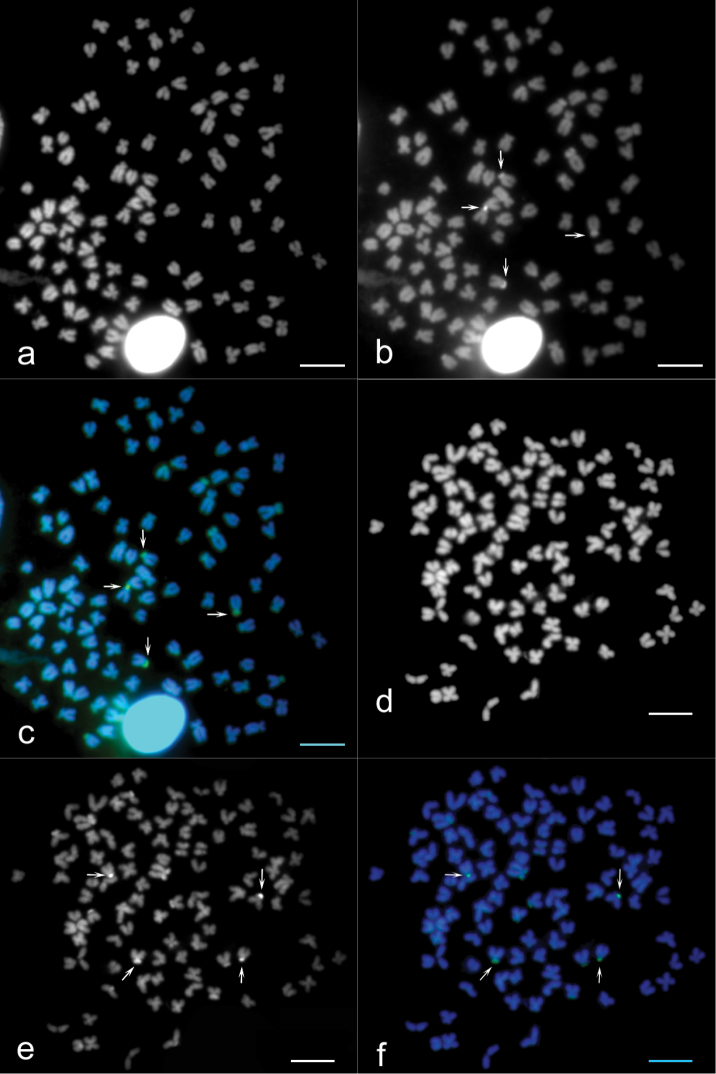
**a–f** Sequential chromosome banding of *Carassius carassius* female chromosomes. Metaphases counterstained by DAPI show all 100 chromosomes (**a**, **d**), metaphases stained by CMA_3_ show 4 NORs (**b, e** white arrows) and the combination of these bandings show 4 identical NORs (**c, f** white arrows; green signals). Bar = 10 μm.

**Figure 3.**
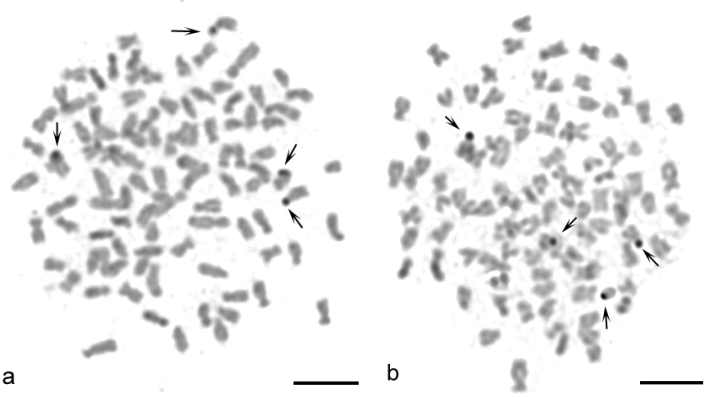
**a–b** AgNOR staining metaphases of *Carassius carassius* female (**a, b** black arrows) indicate 4 NOR-positive sites. Bar = 10 μm.

**Figure 4.**
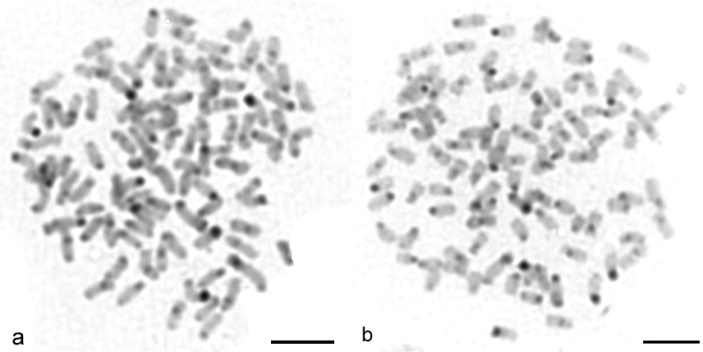
**a–b** C-banded metaphases of *Carassius carassius* female (**a, b**) show signals localized in the telocemeric and pericentromeric chromosome regions. Bar = 10 μm.

**Figure 5.**
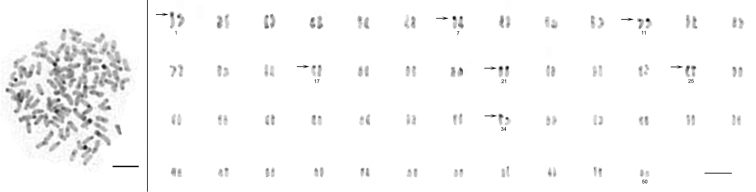
karyotype of *Carassius carassius* female arranged from C-banded chromosomes. Seven pairs of chromosomes show significant signals (black arrows). Bar = 10 μm.

Sequential banding (DAPI + CMA_3_) revealed four CMA_3_-positive bands situated at the sites of the secondary constrictions on the p arms of the 14^th^ pair of sm chromosomes and on the p arms of the 32^nd^ pair of st-a chromosomes (Figs 2b, c, e, f). DAPI uniformly stained all chromosomes (Figs 2a, d). AgNOR analysis revealed four positive signals (Figs 3a, b) which corresponded to four CMA_3_ positive signals. C-banding detected blocks of constitutive heterochromatin at the telomeric and pericentromeric chromosome regions (Figs 4a, b). Telomeric signals were more intensive than pericentromeric ones. C-banded chromosomes were arranged in an karyotype (Fig. 5). Seven chromosome pairs had conspicuous C-banded arms.

## Discussion

The karyotype of all the five individuals of crucian carp from Byšičky ox-bow had the same diploid chromosome number 2n = 100. This number equalled the value reported in other previous studies (Table 1) except those by Raicu et al. (1981) and Chiarelli et al. (1969). Interestingly, Raicu et al. (1981) found the diploid chromosome number 2n = 50 in individuals from the Danube Delta. Although this report might be a result of a laboratory-generated error (slide mix-up), our closer inspection of the published karyotype did not provide any obvious answer. Vasilev and Vasileva (1985) discussed the finding of Raicu et al. (1981) and suggested that the presented karyotype belonged to a member of the genus *Gobio* Cuvier, 1816. At present, it is difficult to speculate more about the observed difference between the reported chromosome numbers unless detailed population screening of this species will be available. In contrast to the results obtained by Raicu et al. (1981), the diploid number of 104 chromosomes presented by Chiarelli et al. (1969) could be most likely attributed to preparation artifact.

The present study demonstrated that karyotype of individuals of *Carassius carassius* under study possessed 10 pairs of metacentric, 18 pairs of submetacentric and 22 pairs of subtelo- to acrocentric chromosomes, already reported by Knytl et al. (2013) as a haploid component of the genome of the allopolyploid female of *Carassius gibelio*. Arrangement of chromosomes within the karyotype was different compared with other findings (i.e. Hafez et al. 1978, Sofradžija et al. 1978), probably due to a different level of chromosome spiralization (Ráb and Collares-Pereira 1995). Two other available studies dealing with the number, location and chromosomal characteristics of the major rDNA sites (Mayr et al. 1986, Boroń et al. 2010) showed four chromosomal sites on two different sm pairs of chromosomes. We also observed this pattern, i.e. four mutually corresponding CMA_3_ and AgNOR signals respectively, on the secondary constrictions on the short arms of a single pair of sm chromosomes and another pair of st-a chromosomes. Though this chromosomal pattern is very common, it represents an additional evidence in favor of paleotetraploidy of the crucian carp genome as suggested by Vasilev and Vasileva (1985). This hypothesis must be examined using other techniques, since it was proven in other similar cases when common carp *Cyprinus carpio* (Larhammar and Risinger 1994, David et al. 2003, Zhang et al. 2008) as well as various species of *Barbus* Cuvier, 1816 (*sensu lato*) (Chenuil et al. 1999) were also revealed as evolutionary tetraploids based on sequences and substitutions analyses, as well as microsatellite analyses respectively.

DAPI-counterstained chromosomes did not provide any useful information since the observed signals were uniform throughout the chromosomes. Similar results were reported for *Carassius gibelio* by Zhu and Gui (2007).

We have performed C-banding on chromosomes of *Carassius carassius* for the first time. Constitutive heterochromatin blocks detected by C-banding method were located in telomeric regions of 7 pairs of chromosomes. Number of these signals can be a species-specific marker, especially in paleotetraploid forms.

Although there is no information about sex differences between *Carassius carassius* karyotypes, we have to point out that only one male specimen was included in this study

In respect to its status of a highly endangered fish species and unclear distribution of possible diploid and/or paleotetraploid forms as well as ongoing hybridization process with other species of this genus across its range of distribution, the present study is a moderate but important contribution to the cytogenetics and cytotaxonomy of *Carassius carassius*.
